# The Relationship between Functional Health Literacy, Self-Rated Health, and Social Support between Younger and Older Adults in Ghana

**DOI:** 10.3390/ijerph16173188

**Published:** 2019-08-31

**Authors:** Padmore Adusei Amoah

**Affiliations:** School of Graduate Studies; Asia Pacific Institute of Ageing Studies; Centre for Social Policy & Social Change, Lingnan University, Hong Kong (SAR); pamoah@LN.edu.hk or

**Keywords:** health literacy, social support, social networks, health status, young and emerging adults, older persons, Ghana

## Abstract

It is well established that health literacy positively affects health outcomes, and social support influences this association. What remains unclear is which aspect of social support (instrumental, informational, and emotional support) is responsible for this effect and whether the influence differs from one population group to another. This study addresses these lacunae. It examines the impact each type of support makes on the relation between functional health literacy (FHL) and self-rated health status among younger and older adults in Ghana. Data were pooled from two cross-sectional surveys, together comprising 521 participants in the Ashanti Region. The results indicated that young adults were more likely to possess sufficient FHL and perceive their health more positively than older adults. While FHL was positively associated with health status, the relation was stronger when young adults received a high level of emotional support. Among older persons, informational support substantially moderated the association between FHL and health status. Thus, social support modifies the relations between FHL and health status among younger and older adults in different ways and to different degrees. Therefore, interventions to improve FHL and health amongst younger and older adults should pay due regard to relevant aspects of social support.

## 1. Introduction

Health literacy refers to the “cognitive and social skills which determine the motivation and ability of individuals to gain access to, understand and use information in ways which promote and maintain good health” [1]. It is considered a robust determinant of population health and well-being among both younger and older cohorts of populations [2,3]. Limited health literacy is associated with poor health, higher healthcare costs, higher rates of hospitalization, and limited use of preventive health services [1,4,5]. For instance, in a study among older persons in the United States, Baker et al. [6] observed that adequate health literacy was associated with low mortality; while research among some youth in Ghana also found that persons with low health literacy are less likely to perceive their health in favorable terms [7,8]. Therefore, some scholars have theorized a causal relationship between health literacy and health [2]. Studies show that health literacy is determined by a labyrinth of individual and environmental factors. Factors such as educational level, cultural and social norms, the health system, and the interactions among them determine health literacy and its influence on health [1,2,9]. Nonetheless, conceptual and empirical approaches to understanding the nature of the relationship between health literacy and health, and the factors that explain and influence it, are still evolving and require cross-national evidence and validation to support existing efforts [1]. Therefore, this article compares the moderating influence of different forms of social support in the well-established positive relationship between health literacy (functional health literacy, FHL) and self-rated health status among younger and older adults in Ghana. FHL is an aspect of health literacy and entails the understanding required to adhere to medication regimen and to comply with warnings about health-deteriorating activities [1]. This fundamental aspect of health literacy was chosen for investigation in this study in view of the limited research previously undertaken in Ghana, and particularly among older adults. 

Recent research strongly links disparities in health outcomes to social support [10,11,12,13] and its influence on health literacy [14,15,16]. However, these studies have not adequately considered the age dynamics of its influence—i.e., how social support affects the relations between health literacy and health status of younger and older adults. Social support refers to “the network of family friends, neighbors and community members that is available in times of need to give psychological, physical, financial, or other kinds of help” [17]. Others consider it “a coping resource—a social fund from which people may draw when handling stressors” [18]. To many, it is a critical component of social capital—social relationships and the resources embedded in them [19]. Social support, therefore, comprises two main components: the network aspect and the functional aspect [18,19]. The elements constituting the functional aspect, which are the focus of this paper, can be categorised into three major types of support, namely emotional support (helping others to feel things; e.g., love, care, sympathy, understanding, and values), instrumental/tangible support (helping others to do things; e.g., household chores, money, and transportation), and informational support (helping others to know things) that one gains through diverse social networks [17,19]. While social support can have a positive effect on health, there are several actual and conceivable instances where it does and can generate adverse effects such as exerting unreasonable pressure, providing misinformation, introducing stigma, and judgmental attitudes, which lead to physical and psychological distress [20,21]. Nonetheless, research shows that social support is crucial for transmitting appropriate health information and monitoring behaviors and practices among all population groups [22,23,24]. Such practices are known to promote good health practices, even among those with chronic ailments [11,25]. 

### Social Support, Health Literacy, and Age

Consideration of age as an indicator of health is common [26]. For instance, younger age is often associated with “fitness, energy and strength” [26]. It is a stage where “the slow deterioration of health associated with adulthood has yet to take hold; childhood illness and vulnerabilities have been left behind” [26]. Therefore, as regards social support, studies suggest that the need for it, and its availability vary with age [17]. Hence, a study in northern Greece found that social support was more strongly associated with health and well-being of older persons than it was to young adults [27]. However, observation in other places demonstrates inconsistencies in the effect of social support on health across age groups and genders [28,29]. In Ghana, much has been written about the crucial role of social resources in the health of different age groups and conditions [12,13,30,31]. This influence is widespread among older persons who are known to rely heavily on their social networks for all forms of support (including access to healthcare), as the majority of them are poor and live in deprived communities [12,13,32,33]. Indeed, traditionally, the family has served as the primary provider of material and intangible support to older persons in Ghana [34]. Although low health literacy is seen as one of the causes of poor health in Ghana, little research has touched on how prevailing social resources affect health literacy. Related studies have concentrated on general adult populations, pregnant women, and gender [35,36,37]. Therefore, the role of social support in how FHL affects the health of younger and older persons remains poorly understood and the situation begs the question of which, and how, different aspects of social support influence health among various population groups? Thus, by tackling this question, this paper addresses the longstanding theoretical and empirical research gaps by pinpointing how social support works to sustain or improve the health and well-being of different age groups [23].

Results from research that has been conducted on the influence of social factors such as age, level of education, gender, and marriage on the relationship between health literacy and health status vary. A substantial body of evidence indicates that younger persons are more likely to have adequate FHL as they tend to be better educated [1,3]. However, the propensity of adequate FHL and health declines with age regardless of educational level, gender, or ethnicity, because aging is associated with weak cognitive abilities [6,38]. Accordingly, social support is critical to the extent to which health literacy, particularly FHL, influences health [24,39,40,41,42]. For instance, in a population-based study in South Korea, Kim et al. [40] observed that social support emanating from even weak social ties moderated the relations between FHL and health information efficacy (ability to perform tasks such as finding, processing, and understanding health information). Likewise, research among older persons in the USA found that social support enhances the association between FHL and health, even among persons with adequate health literacy [41]. 

As regards the distinct social support proxies, there are compelling indications that extensive access to informational support enhances FHL and promotes participation in medical decision-making [43,44]. Older persons with low FHL tend to require more informational support compared with younger persons [44]. Emotional support is also known to shape positive attitudes towards health decisions, especially among people with low literacy skills and educational attainment, as well as those facing various livelihood stressors, such as young people [26,43]. Furthermore, in many settings, reliance on social ties for tangible assistance, such as money and transportation for the sake of health and healthcare, are common, although sometimes subtle [14,15,31,44]. A study of people with low socioeconomic statuses and depressive symptoms found that instrumental support independently mediates the association between FHL and health status [15]. However, social support may not always be necessary for health literacy, as both younger and older adults with low FHL often feel ashamed and distanced from their peers, families, and even health professionals [45,46]. Putting the above evidence together, it is hypothesized that each of the social support proxies will positively moderate the relations between FHL and health. Additionally, the role of social support for younger and older adults will differ, owing to variations in FHL and social support needs. This assertion is largely supported by the tenets of the broader ecological theory of aging [47,48]. The theory recognizes that age-related cognitive and physical frailty is inevitable, and measures are required to strengthen the agency and sense of belonging to enable people to manage their environments (including health services, information, and the health system in general) successfully as they age [47,48]. Social support is considered influential in this process but may function differently for various population and age groups, depending on their physical status, cognitive and social skills, and economic conditions [15,39,48]. As regards health literacy, its influence on health outcomes is said to be embedded in the social environment [2,37,40,49]. Thus, despite the apparent inconsistencies in current evidence, social support is likely to positively affect the relations between FHL and health status, especially among older persons who are expected to have low FHL. 

## 2. Methods 

### 2.1. Study Design and Data Collection

The data for this paper were pooled from a survey on Social Capital, Health Literacy, Access to Healthcare, and Health Research, which was conducted on two occasions among adult populations (18 years or above), using a cross-sectional design, in Ghana. The data were collected from June to October 2015 and between January and July 2017 in the Ashanti Region in Ghana. The Region is the most populous in the country, partly due to its nodal location. This paper analyzes data from 521 participants, who were included from both surveys based on specified age criteria; younger and emerging adults (ages 18–29 years) [50], and older adults (50 years and above). The age limit for older persons took into account the low life expectancy in Ghana [51] and the fact that “old age” is a product of social perception and economic necessity [52]. Previous studies in Ghana have adopted a similar age limit [12]. Overall, 417 cases (out of 779) were relevant from the first survey, and an additional 104 (out of 223) was applicable from the second survey. The primary sample was statistically over the minimum required sample, and the deduced sample provided adequate data to conduct the analysis. Based on the recommended formula for determining overfitted models; 50 + 8*n* (where *n* is the number of predictors) [53], a minimum sample of 130 cases each was required for the younger and older adults. However, this study included 318 younger and emerging adults and 203 older persons. These indicate that the models were not overfitted and justify the adequacy of the sample. 

In both surveys, a multistage cluster sampling approach [54] was employed to select specific communities and participants for the study. Participants came from five districts, namely Kumasi Metropolitan Area, Atwima Kwanwoma District, Kwabre East District, Ejisu-Juaben Municipality, and Asokore-Mampong Municipality. Each of these districts presented unique features (including rural or urban characteristics, poverty levels, access to public services, ethnicity, and religious diversity), which altogether represented the study region adequately. Based on these attributes, 53 strata were constructed for the study, including 42 urban suburbs/communities (with 36 from the first survey) and 11 rural communities (with eight from the first survey). The questionnaires were distributed proportionally to the various stratum based on estimated population sizes. A pilot survey had shown that many potential participants could neither read nor understand the questions satisfactorily due to low literacy. Therefore, the questionnaires were interviewer-administered by trained staff who were conversant with the local language (Twi) and prevailing sociocultural dynamics. A fixed interval was used to systematically select participants based on an approximate number of houses in urban suburbs and rural communities. In rural communities, a person from every second house in a stratum was interviewed, whereas a person from every fifth house in urban areas was interviewed due to the differences in community sizes. Each interviewer began from an arbitrarily selected house in different cardinal and intercardinal points of a stratum and moved towards the end of the row. In rural communities, interviewers were usually assigned rows, with each moving in different directions. The process avoided interviewing more than one person from a house, because many residences accommodated several households, and residents often shared similar characteristics and living arrangements. In all the houses, a convenience sampling technique was adopted to interview one person from those who were available and agreed to be included in the study. The research design has also been explained elsewhere [35,37].

### 2.2. Measures

#### 2.2.1. Outcome Variable: Self-Rated Health Status 

Self-rated health status was measured by asking participants to rate the state of their health on a five-point scale as either poor, fair, good, very good, or excellent. This approach has been used successfully across several contexts to measure health status [35].

#### 2.2.2. Independent Variable: FHL 

The study used a slightly modified version of the Swedish Functional Health Literacy Scale (SFHL) to measure FHL. The SFHL is a five-item tool with five response options, namely “never”, “seldom”, “sometimes”, “often”, and “always” [55]. Respondents were asked questions such as: “Do you think that it is difficult to read health information because the text is difficult to see?” And, “do you think that it is difficult to understand the message in health information?” The SFHL was employed because it has been well tested among different population groups and has also been previously used in Ghana [4,55,56]. The tool was adapted to suit the local context after back-translation (English–Twi–English) and pretesting. The response labeled originally as “seldom” was changed to “not often”, and “always” was changed to “all the time”. The new categories were easier to translate into the local language during interviews. The SFHL instrument showed adequate reliability with a Cronbach’s alpha of 0.85. However, it must be emphasized that as the instrument only measures FHL, it can only be presented as adequate rather than a holistic measure of health literacy [56]. 

#### 2.2.3. Moderator: Social Support 

The measurement of the three types of social support was inspired by the Adapted Social Capital Assessment Tool (S-ASCAT) [19,57]. Participants were asked if they had received any support in emotional, instrumental, or informational form, through any of their social networks in the past 12 months. Examples were attached to each type of social support to make them more understandable and distinct. 

#### 2.2.4. Covariates 

The following demographic characteristics of participants were included: age (in years), sex (male and female), and educational attainment (never been to school, basic education, senior high school, and tertiary education). Others included employment status (employed and unemployed including students), average monthly income/stipend (measured in New Ghana Cedis), marital status (married and unmarried), and location of residence (rural or urban).

### 2.3. Analytical Strategy

The analyses comprised of descriptive univariate analysis and ordinal logistic regression using SPSS version 25. The univariate analysis examined the frequencies and differences between younger and older adults concerning their socio-demographic, social support, health literacy, and health status characteristics. The descriptive analysis also included a Spearman’s correlation analysis of the variables in the study (Supplementary Table S1).

In the descriptive analysis, three levels of FHL were derived to provide precise differences in health literacy of younger and older adults. In the process, responses to “never” and “not often” were classified as sufficient FHL. All responses to “sometimes” were labeled as problematic FHL, and “often” and “all the time” were categorized as “inadequate FHL” [55]. For the predictive analysis, a continuous variable of the FHL was used to limit the loss of data. This was derived by summing all the responses to the five items, to have a minimum score of 5 and a maximum of 25. 

In the second part of the analysis, an ordinal logistic regression technique was used to elicit the independent and interaction predictors of self-rated health status. Three models were constructed for younger adults, older adults, and the overall sample. The variables included FHL, the three social support proxies, and the interaction terms between social support (each proxy) and FHL. The models controlled for sociodemographic factors that showed significant relations with self-rated health status in the Spearman’s correlations analysis, which included the year of data collection. All variables were standardized before being included in the analysis to reduce multicollinearity. Four variables— education, income/stipend, age, and emotional support—had an average of 2.3 missing responses, and they were replaced with the mean. Following the regression analysis, the significant interaction results were scrutinized further using the simple slope analysis technique to ascertain the level of the social support proxies that moderate the association between FHL and health status among younger and older adults. The simple slope analysis tested whether high social support (one standard deviation above the mean) or low social support (one standard deviation below the mean) enhanced or attenuated the relation between FHL and health status. The slope analyses used a constant (unstandardized regression coefficient) of 3 to project a better figure (aesthetically) as it did not influence the results in any way [58]. The significance level for all associations was set at *p* < 0.05.

## 3. Results

According to Table 1, most participants were females, and they mostly lived in urban areas. The majority of older persons had never been to school, while the young adults had Junior High School as the commonest educational attainment. Being married was a more common phenomenon among older persons than among young adults. Sufficient FHL was prevalent among young adults, while inadequate FHL was predominant among older adults. The young adults received more emotional support than older adults did. Also, young adults were more likely to perceive their health in favorable terms compared with older adults. Supplementary Tables S2 and S3 contain the descriptive statistics for each of the two datasets used for the study. 

Table 2 shows the results of the ordinal logistic regression analysis. FHL was positively associated with health status among both younger and older adults as well as the overall participants. As regards social support, informational and emotional support were positively associated with the health status of young adults and the overall participants. Only informational support was associated with self-rated health among older adults. In the moderation analysis, emotional support positively modified the relation between FHL and health status among young adults. Informational support also positively moderated the association between FHL and health status, and the same result was observed among the overall participants. Instrumental support, neither independently nor interactively influenced health status in any of the groups. 

**Figure 1:**
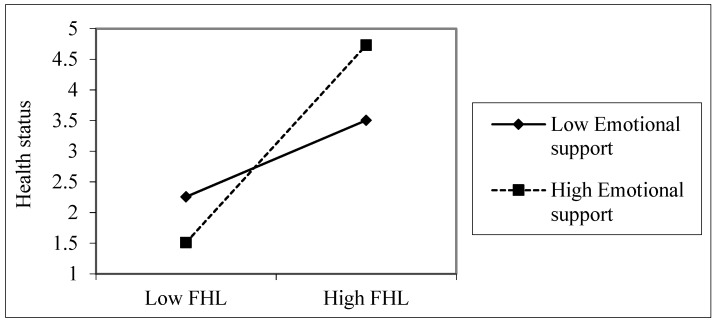
The association between emotional support, FHL, and health status among younger adults. Health status was measured on a five-point scale (poor, fair, good, very good, and excellent). The values used in the plotting are based on the unstandardized regression coefficients.

**Figure 2:**
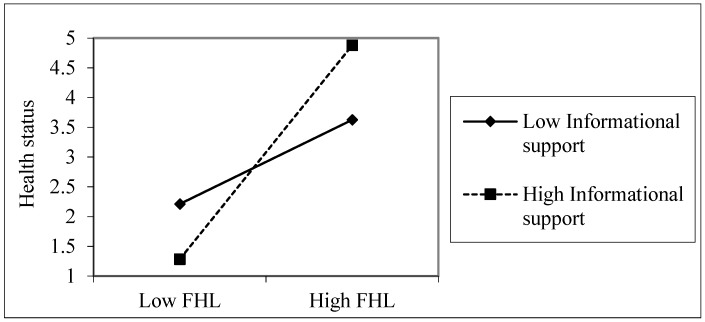
The association between informational support, FHL, and health status among older adults Health status was measured on a five-point scale (poor, fair, good, very good, and excellent). The values used in the plotting are based on the unstandardized regression coefficients.

**Figure 3:**
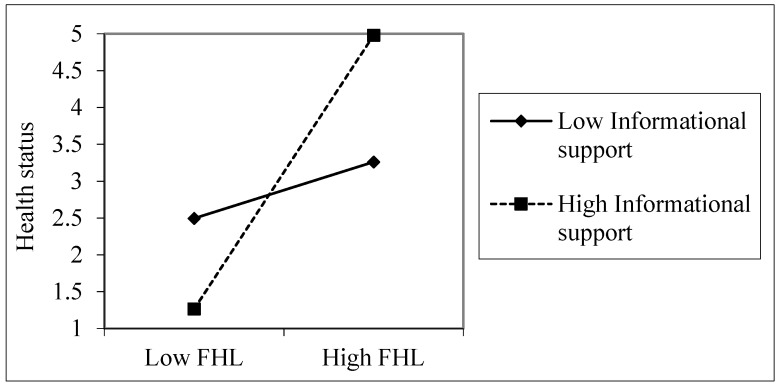
The association between informational support, FHL, and health status, the overall sample. Health status was measured on a five-point scale (poor, fair, good, very good, and excellent). The values used in the plotting are based on the unstandardized regression coefficients.

The simple slope analyses showed that FHL was positively related to health status when emotional support was high (β = 0.424, *t* = 2.949, *p* = 0.003), but FHL was not significantly related to health status at a low level of emotional support among young adults (β = 0.164, *t* = 1.143, *p* = 0.254), as shown in Figure 1. Among older adults, FHL was more useful for health when informational support was high (β = 0.377, *t* = 2.20, *p* = 0.028), compared with when it was low (β = 0.249, *t* = 1.540, *p* = 0.125), as shown in Figure 2. The overall sample also showed a similar pattern (Figure 3). FHL was positively related to health status if a person had high informational support (β = 0.315, *t* = 3.067, *p* = 0.002). The influence of FHL on health status was not significant when informational support was low (β = 0.065, *t* = 0.633, *p* = 0.527). Thus, informational support (among older adults) and emotional support (among young adults) strengthened the association between FHL and health status.

## 4. Discussion

The study investigated whether various kinds of social support moderate the relations between FHL and health status among younger (and emerging) and older adults in Ghana. To the best of my knowledge, this is the first study to explore the moderating influence of social support on FHL and health status among different age groups. The results were mostly consistent with the hypothesis that social support positively moderates the relation between FHL and health status. Also, there were variations in the types of social support which modified this relation among younger and older adults, as anticipated. Besides, sociodemographic characteristics of participants such as sex, area of residence, marital status, and educational attainment were mostly consistent with those of the study area (Ashanti Region) [51]. The results showed that FHL was positively associated with health status for all groups, although young adults were more likely to demonstrate sufficient FHL as compared with older persons. These findings are consistent with prior research suggesting that adequate health literacy enhances health and well-being [3,4,7]. In postmodern societies, younger persons are more likely to attain some formal education, which empowers and encourages them to seek health information for themselves. Indeed, young adults in this study had higher levels of education than the older participants, even though a significant predictive association between education and health literacy was not established in this paper. Moreover, existing research has found a positive connection between youth and higher levels of cognitive function, which enables younger people to obtain and adopt knowledge from a greater variety of sources [38]. Younger adults are also more careful and concerned about their bodies, demonstrate awareness of their health needs, and attempt to emulate healthy lives consistently [26]. These youth-related concerns explain why the participants possessed sufficient FHL and professed better health outcomes as compared with older persons in the present study. 

Young adults received more emotional social support from their social networks than older adults. Correspondingly, emotional support moderated the relation between FHL and health status among young adults, but its effect was not significant among the older persons. This adds to existing evidence of the complexity and dynamism of the concept of social support. The significance of emotional support among young persons can be explained from two perspectives. First, dependence on parents/guardians and older adults for both social and economic support is a commonplace phenomenon in settings such as Ghana, due to inadequate opportunities, especially in areas of employment in Ghana [59]. Therefore, it comes as no surprise that only high levels of emotional support—which is intangible—positively moderated the relations between FHL and health status among young adults. Even though younger adults are more likely to have a sufficient level of FHL than older adults, health-related issues, particularly those of “emotional nature, characterise the lives of a significant minority of young people” (West, 2009 in [26]), and require appropriate health promotion strategies in order to identify them in practice. Second, prior research shows that people with inadequate FHL tend to experience shame or guilt, and even attempt to hide their deficiencies from close friends and family [15,45,60]. This suggests that emotional support is an essential element in addressing the stigma attached to low FHL. Without it, young adults are likely to be stuck in their confusion by not opening up to others about their health-related limitations, which may eventually have dire consequences for their health [15]. Observations from previous studies suggest that elements of social support produce a significant sense of purpose in life, even for older persons [61]. In fact, some argue that “social support is what helps us maintain our sociability, persist in our goals and resist isolation and despair” [17]. Such an assertion is linked to emotional support based on its differing, but significant, effect on FHL and health for both young adults in this study. In part, these reasons explain why many young adults may require high emotional support to enable them to demonstrate and apply their innate and learned competencies, including FHL for better health outcomes. 

The results also revealed that high informational support modified the extent of the influence that FHL has on health status among older adults. The effect of FHL on health status was insignificant at low levels of informational support. Older adults may frequently solicit and heed the information delivered through their social networks, which enhances their competencies about health and prepares them to interact productively with the health system and the broader environment, in line with the tenets of the broader ecological theory of aging [47,62]. In the age of technological advancement, it is conceivable that older persons are likely to rely on their social environments to access and use new information retrieval systems to exert their agency and adapt to their environments [47]. Indeed, older persons are more likely to depend on their social networks to seek and apply health information, unlike younger adults who are usually educated and more capable of navigating their health systems for themselves [38,44]. For instance, Lee et al. [44] found in their study in the USA that older persons with low FHL are more likely to receive informational support. This finding compares favorably with that of Edwards et al. [63], who also discovered in South Wales in the UK that people in frail conditions actively rely on their social networks for support in healthcare decision-making and communicating with health professionals. The results of the slope analysis can amply demonstrate these. In all the observed significant associations in the present study, high social support moderated the relations between FHL and health status. Thus, FHL is more likely to affect health status only in conditions where people had adequate social support—particularly emotional and informational support.

Moreover, it was surprising to note that instrumental support was not associated with health; neither did it moderate the effect of FHL on health status for either younger or older adults. Many in Ghana consciously depend on their social networks for pecuniary assistance and other kinds of support to access health services [12,31]. Thus, one would have expected this form of social support to make a significant impact on health literacy and health outcomes given its visible role in empowering people to change their environments [47]. However, Stewart et al. [15] found in a related study that while instrumental support mediated the relations between health literacy and health independently, it failed to do so in a multivariate analysis. This suggests that instrumental social support works through other types of social support, such as emotional and informational support, as has been observed in another work in Ghana [49]. Thus, the role of instrumental support in FHL may take a more nuanced and indirect form. This explains why Brabers et al. [43] excluded this form of social support in their study in the Netherlands. Also, other studies contend that despite its essential role in the health status of older persons, instrumental support is not often featured in interventions to address the incidence of low FHL [44].

While the study illuminates the role of social support in the relation between FHL and health status, the findings should be interpreted with some caution. First, the data were pooled from a cross-sectional study, and therefore, the data cannot account for causality. Second, the study used instruments which elicit self-efficacy, such as the self-rated health status and FHL. Therefore, some participants may have responded in a manner that was considered socially desirable instead of reporting their actual conditions. Finally, the study relied on a relatively small sample size for the two subgroups. Thus, one could contest the robustness of the results. Nonetheless, as argued above, the sample was adequate in view of the nature of the models constructed [64,65]. Furthermore, and as prompted by Gelman and Carlin [66], the results are consistent with extant literature on relations among health literacy, social support, and health [3,14,15,16]. 

## 5. Conclusions

The study examined the role that social support plays in the interaction between FHL and health status among younger and older adults in Ghana. Different social support proxies, such as emotional and informational support, moderated the relation between FHL and health status among younger and older adults in different ways. Therefore, emerging from this study, and critical to health promotion strategies, is the need to account for relevant aspects of social resources in policy and practice relating to different age groups. Health education interventions should be carried out with empathy and care by involving the social networks of younger adults. Among older adults, the social support systems can be conduits for delivering proper health information because their role in health literacy, and by extension, health, is cardinal. Future research should extend this study into an examination of the nuances of these relations using qualitative approaches to provide in-depth knowledge, and especially explain why instrumental support does not modify the influence of FHL on health status. 

## Figures and Tables

**Table 1:** Sociodemographic characteristics of participants.

	Young and Emerging Adults 18–29 years, N = 318	Older Adults 50+ years, N = 203		Overall (N = 521)
	%	%	*p-value ^a^*	N (%)
**Sex**			0.266	
Male	44.3	49.3		46.3
Female	55.7	50.7		53.7
**Context/People**			0.993	
Rural	38.9	39.0		45.3
Urban	61.1	61.0		54.7
**Educational Attainment**			**0.001**	
Never been to school	0.6	50.2		20.0
Basic education (Junior high school)	48.8	31.0		41.3
Senior High School (SHS)	44.0	12.3		32.1
Tertiary Level	7.5	5.4		6.7
**Marital Status**			**0.001**	
Married	20.1	51.2		48.8
Unmarried	79.9	48.8		51.2
**Employment Status**			0.884	
Employed	51.9	51.2		51.6
Unemployed	48.1	48.8		48.4
**Monthly Income/Stipend ^b^**			**0.001 ^c^**	
<200 GH¢	52.2	64.9		58.5
200–500 GH¢	26.7	14.9		20.9
500–1000 GH¢	16.1	13.2		14.7
1000+ GH¢	5	6.9		5.9
**Informational support**			0.416	
No	25.5	28.6		26.7
Yes	74.5	71.4		73.3
**Instrumental support**			0.573	
No	24.5	26.7		25.3
Yes	75.5	73.3		74.7
**Emotional support**			**0.033**	
No	22.3	30.7		25.6
Yes	77.7	69.3		74.4
**Health literacy**			**0.001**	
Sufficient HL	43.1	28.1		37.2
Problematic HL	35.5	21.7		30.1
Inadequate HL	21.4	50.2		32.6
*Mean (SD)/ Minimum-maximum ^d^*	*19.2 (3.8)/5–25*	*15.6 (5.1)/5–25*		*17.8 (4.9)/ 5–25*
**Health status**			**0.001 ^c^**	
Poor	10.1	15.3		12.1
Fair	15.1	22.2		17.9
Good	26.7	31.5		28.6
Very good	32.7	25.1		29.8
Excellent	15.4	5.9		11.7

^a^ Bold figures = *p* < 0.05. **^b^** 1US$ = GH¢ 3.8 and 4.3 in 2015 and 2017, respectively, GH¢ = New Ghana Cedis. ^c^ *p*-values are based on independent samples test, and all others are based on Chi-square tests. ^d^ Mean, SD (standard deviation), and minimum and maximum values are based on the sum of all responses to the SFHL (Swedish Functional Health Literacy Scale) instrument.

**Table 2:** The relationship between social support proxies and health literacy and health status by ordinal logistic regression ^b^.

	Young and Emerging Adults (18–29 years)				Older-Persons (50 years +)				Overall			
	Estimate (B)	Std. Error	95% Confidence Interval		Estimate (B)	Std. Error	95% Confidence Interval (B)		Estimate	Std. Error	95% Confidence Interval (B)	
			Lower Bound	Upper Bound			Lower Bound	Upper Bound			Lower Bound	Upper Bound
**Age**	−0.530	0.521	−1.551	0.490	−0.097	0.141	−0.373	0.180	−0.270 *	0.116	−0.498	−0.043
**Income/stipend**	0.144	0.212	−0.271	0.559	0.258 *	0.125	0.013	0.504	0.223 *	0.098	0.031	0.414
**Marital status**												
Not married (ref)												
Married	0.664 **	0.282	0.111	1.217	0.177	1.003	−2.143	1.788	0.504 *	0.235	0.044	0.964
**Information support**												
No (ref)												
Yes	0.247 *	0.102	0.008	0.485	0.184 *	0.061	0.019	0.421	0.278 **	0.097	0.087	0.467
**Instrumental support**												
No (ref)												
Yes	−0.169	0.123	−0.410	0.071	0.114	0.196	−0.260	0.488	−0.063	0.097	−0.254	0.127
**Emotional support**												
No (ref)												
Yes	0.286 **	0.106	0.058	0.513	0.150	0.194	−0.231	0.531	0.166 *	0.043	0.026	0.349
**Health literacy**	0.294 **	0.099	0.081	0.478	0.261 *	0.163	0.059	0.580	0.190 *	0.050	0.035	0.376
Health literacy * Instrumental support	−0.005	0.127	−0.255	0.244	0.077	0.171	−0.257	0.412	−0.040	0.096	−0.228	0.148
Health literacy*Informational support	0.193	0.132	−0.451	0.065	0.238 *	0.053	0.072	0.531	0.284 **	0.089	0.109	0.459
Health literacy*Emotional support	0.309 **	0.121	0.071	0.547	0.151	0.143	−0.129	0.431	0.117	0.091	−0.062	0.296
*Cox and Snell*	*0.086*				*0.117*				*0.175*			
*Nagelkerke*	*0.091*				*0.123*				*0.178*			

Notes: N = 521 for overall, 318 for young adults and 203 for older persons; *** Significant at the 0.001 level (2-tailed), ** Significant at the 0.01 level (2-tailed), * Significant at the 0.05 level (2-tailed). B = unstandardized coefficients. ^b^ The models also controlled for the year of data collection (i.e., 2015 and 2017).

## Supplementary Materials

The following are available online at https://www.mdpi.com/1660-4601/16/17/3188/s1: Table S1 (Spearman’s correlation analysis of variables in the study); Table S2 (Descriptive statistics of first data (June to October 2015); and Table S3 (Descriptive statistics of second data (January to July 2017).
