# Supplementary material for: The Relationship between Functional Health Literacy, Self-Rated Health, and Social Support between Younger and Older Adults in Ghana
Source: Int J Environ Res Public Health. 2019 Aug 31;16(17):3188. doi: 10.3390/ijerph16173188 (PMC6747074; doi:10.3390/ijerph16173188)
Supplement: Supplementary file 1 [file ijerph-16-03188-s001.pdf]

Supplementary Table S1: Spearman's correlation analysis of variables in the study.

		1	2	3	4	5	6	7	8	9	10	11	12	13
1.	Health status	1.000												
2.	Information support	0.128*	1.000											
3.	Instrumental support	-0.083	0.513**	1.000										
4.	Emotional support	0.102*	0.389**	.460**	1.000									
5.	Health literacy	0.168**	0.439**	0.491**	0.406**	1.000								
6.	Age	-0.140**	0.460**	0.482**	0.429**	-0.704**	1.000							
7.	Sex	-0.018	-0.028	0.036	0.085	0.091*	-0.057	1.000						
8.	Locality (Rural)	-0.060	0.016	-0.091*	0.087*	-0.096*	0.063	0.179**	1.000					
9.	Marital status	0.193**	0.473**	0.453**	0.400**	0.777**	-0.757**	0.064	0.057	1.000				
10.	Education	0.048	0.247**	-0.264**	-0.210**	0.175**	-0.394**	-0.080	-0.201**	0.333**	1.000			
11.	Income	0.225**	0.443**	0.470**	0.350**	0.768**	-0.648**	0.090	0.039	0.682**	0.450**	1.000		
12.	Employment status (employed)	0.059	0.370**	0.368**	0.285**	0.608**	0.657**	0.085	0.050	0.623**	0.253**	0.415**	1.000	
13.	Year of data collection	-0.101*	0.051	0.059	0.066	0.025	0.045	-0.009	0.173**	-0.028	-0.068	-0.053	-0.111**	1.00

***Significant at the 0.01 level (2-tailed), *Significant at the 0.05 level (2-tailed)*

Supplementary Table S2: Descriptive statistics of first data (June to October 2015)

	Young and emerging adults 18-29 years, N= 255		Older adults 50+ years, N = 162			Overall (N=417)
	Frequency	%	Frequency	%	*p-value*	N (%)
**Sex**					**0.042**	
Male	114	44.7	79	48.8		193 (46.3)
Female	141	55.3	83	51.2		224 (53.7)
**Context/People**					0.841	
Rural	121	47.5	77	47.5		198 (47.7)
Urban	134	52.5	85	52.5		219 (52.3)
**Educational Attainment**					**0.001**	
Never been to school	2	0.8	83	51.2		85
Basic education (Junior high school)	121	47.5	52	32.1		173
Senior High School (SHS)	113	44.3	19	11.7		128
Tertiary Level	19	7.5	8	4.9		25
**Marital Status**					**0.001**	
Married	50	19.6	85	52.5		135 (32.4)
Unmarried	205	80.4	77	47.5		282 (67.6)
**Employment Status**					0.616	
Employed	134	52.5	84	51.9		218 (52.3)
Unemployed	121	47.5	78	48.1		199 (47.7)
**Monthly Income** [c]					**0.001**	
<200 GH¢	73	51.0	98	70.0		171 (60.4)
200-500 GH¢	39	27.7	18	12.9		57 (20.1)
500-1000 GH¢	24	16.8	16	11.4		40 (14.1)
1000+ GH¢	7	4.9	8	5.7		15 (5.3)
**Informational support**					.0911	
No	66	25.9	46	28.4		112 (26.5)
Yes	189	74.1	116	71.6		305 (73.1)
**Instrumental support**					0.997	
No	63	24.7	41	25.3		104 (24.9)
Yes	192	75.3	121	74.7		313 (75.6)
**Emotional support**					**0.036**	
No	55	21.6	49	30.2		104 (24.9)
Yes	200	78.4	113	69.8		313 (75.6)
**Health literacy**					**0.001**	
Sufficient HL	104	40.8	45	27.8		149 (46.9)
Problematic HL	92	36.1	34	21.0		126 (30.2)
Inadequate HL	59	23.1	83	51.2		144 (34.5)
**Health status**					**0.001**	
Good	188	73.7	61	37.7		249 (59.7)
Poor	66	26.3	101	62.3		167 (40.3)

Supplementary Table S3: Descriptive statistics of second data (January to July 2017).

	Young and emerging adults 18-29 years, N= 63		Older adults 50+ years, N = 41			Overall (N=104)
	**Frequency**	**%**	**Frequency**	**%**	***p-value***	**N (%)**
**Sex**					0.108	
Male	27	42.9	21	51.2		48 (46.2)
Female	36	57.1	20	48.8		56 (53.8)
**Context/People**					0.428	
Rural	23	36.5	19	46.3		42(40.4)
Urban	40	63.5	22	53.7		62(59.6)
**Educational Attainment**					**0.001**	
Never been to school	0	0	19	46.3		19(18.3)
Basic education (Junior high school)	31	49.2	11	26.8		42(40.3)
Senior High School (SHS)	27	42.9	8	19.6		35(33.7)
Tertiary Level	5	7.9	3	7.3		8(7.7)
**Marital Status**					**0.001**	
Married	14	22.2	19	46.3		33 (31.7)
Unmarried	49	77.8	22	53.7		71(68.3)
**Employment Status**					0.999	()
Employed	31	49.2	20	48.8		51(49.0)
Unemployed	32	50.8	21	51.2		53(51.0)
**Monthly Income** [c]					**0.008**	
<200 GH¢	21	56.8	15	44.1		36(50.1)
200-500 GH¢	9	24.3	8	23.5		17(23.9)
500-1000 GH¢	5	13.5	7	20.6		12(16.9)
1000+ GH¢	2	5.4	4	11.8		6(8.5)
**Informational support**					0.648	
No	15	23.8	12	29.3		27(26.0)
Yes	48	76.2	29	70.7		77(74.0)
**Instrumental support**					0.867	
No	15	23.8	13	31.7		28(26.9)
Yes	48	76.2	28	68.3		76(73.1)
**Emotional support**					0.141	
No	16	25.4	16	39.0		32(30.8)
Yes	47	74.6	25	61.0		72(69.2)
**Health literacy**					**0.001**	
Sufficient HL	33	52.4	12	29.3		45(43.3)
Problematic HL	21	33.3	10	24.4		31(29.8)
Inadequate HL	9	14.3	19	46.3		28(26.3)
**Health status**					**0.001**	
Good	49	77.8	15	36.6		64(61.5)
Poor	14	22.2	26	63.7		40(38.5)
